# Is The Value of FDG PET/CT In Evaluating Renal Metastasis Underestimated? A Case Report And Review of The Literature

**DOI:** 10.4274/Mirt.130

**Published:** 2013-12-10

**Authors:** Mustafa Aras, Fuat Dede, Tunç Önes, Sabahat İnanır, Tanju Yusuf Erdil, Halil Turgut Turoğlu

**Affiliations:** 1 Marmara University School of Medicine, Department of Nuclear Medicine, Istanbul, Turkey

**Keywords:** Akciğer tümörleri, böbrek tümörleri, pozitron emisyon tomografi, fluorodeoksiglukoz F18, bilgisayarlı tomografi

## Abstract

Contrast-enhanced CT or MRI are used as a gold standard imaging modalities in the detection and characterization of renal masses. On the other hand, the role of FDG PET/CT in evaluating primary or metastatic cancers of the kidney is limited due to the excretion of FDG through urinary tract. We reported the FDG PET/CT of a lung cancer patient with multiple metastases in both kidneys which were missed in previous PET/CT, and underestimated on sequential diagnostic abdominal CT study.

**Conflict of interest:**None declared.

## INTRODUCTION

The Kidney is the fifth most common site of metastasis in the body (1). Renal metastases are present in approximately 10% to 20% of cancer patients depending on tumor type and most commonly seen in the setting of other metastatic diseases (2). They are usually asymptomatic and discovered incidentally on imaging studies performed for other purposes or at post-mortem examination (2,3,4,5). Contrast-enhanced CT or MRI is widely used in the detection and characterization of renal masses. 18F-FDG PET/CT is extensively used in oncology, but its utilization in evaluating primary or metastatic involvement of the kidney is limited, because physiological excretion of 18F-FDG through urinary tract may interfere with tumor imaging. Herein we report the 18F-FDG PET/CT of a lung cancer patient with multiple bone and soft tissue metastases including both kidneys which were missed in the previous PET/CT study, and underestimated on the sequential abdominal CT study.

## CASE REPORT

**Figure 1:**
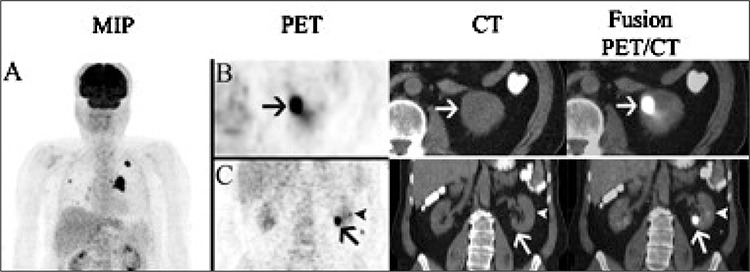
In baseline PET/CT, besides thoracic findings, multiple hypermetabolic bone and soft tissue lesions were observed (A). Although not reported, 2cortical lesions were seen in the lower pole (B-C, arrows) and middle zone (C-D, arrow heads) of the left kidney

**Figure 2:**
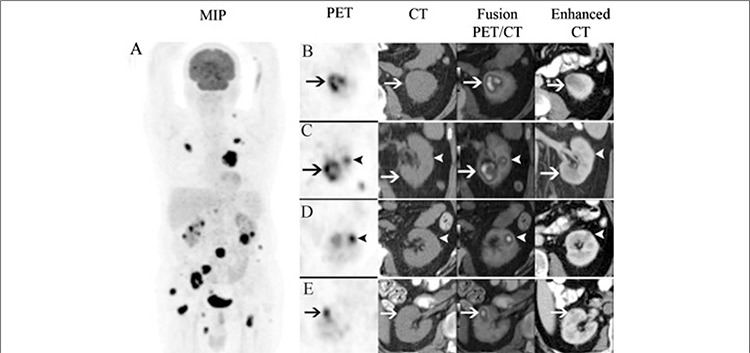
Follow–up PET/CT study showed morpho-metabolic progression compared to initial PET/CT scan (A). The size of the cortical lesion in the lowerpole of the left kidney was increased and the center of the lesion became necrotic (B-C, arrows). The lesion in the middle zone was enlarged and becamemore prominent (C-D, arrow heads). The right kidney was also involved in the current study (E, arrows). Although the sequential i.v. contrast enhanced CTscan showed identical lesions with high image contrast, only predominant left lower pole lesion was reported as a single kidney metastasis

62-year-old male patient with history of non-small cell lung cancer was referred to 18F-FDG PET/CT for initial staging. In baseline PET/CT, besides thoracic findings, multiple hypermetabolic bone and soft tissue lesions were observed, but no lesion was mentioned in kidneys (Figure 1). After three months from the initial staging PET/CT, follow-up contrast enhanced CT imaging including neck, thorax and abdomen was requested. A hypodense lesion in the inferior pole of the left kidney measured 25 mm in diameter was the only additional finding and interestingly no soft tissue involvement was reported on these CT scans. Subsequently, the patient was referred to our university hospital PET/CT unit to evaluate response to treatment. According to our department protocol we used oral but not i.v. contrast agent in routine practice. In the follow–up PET/CT study, in addition to progression in the soft tissue and bone lesions, new foci were found in the cortex of the both kidneys with the largest in the lower pole of the left kidney (Figure 2). At the beginning of the reporting session, the finding of multiple hypermetabolic foci in both kidneys was assumed as physiological urinary retention in the calyceal systems. However, when carefully examined, these hypermetabolic lesions clearly matched with the cortical hypodense lesions on recent contrast enhanced CT images. Interestingly, although the recent i.v. contrast enhanced CT scan showed identical lesions with high image contrast, only predominant left lower pole lesion was reported as a single kidney metastasis (Figure 2). When we reviewed the previous PET/CT for the evaluation of these new lesions, multiple unreported hypermetabolic but less prominent cortical lesions were seen in the left kidney (Figure 1). 

## DISCUSSION

A renal metastasis is the most common malignant neoplasm of the kidney found at autopsy (4). Excluding lymphoma and leukemia, the most common primary sites for renal metastases include lung, colon and breast carcinoma, melanoma and reproductive system malignancies (2,5,6). Metastasis to the kidney usually represents hematogenous spread of the primary malignancies. When renal metastases are detected, the disease has already been disseminated throughout the body. In our case besides thorax and kidneys, moderate to intense hypermetabolic lesions were seen in the skeleton and soft tissue. In the presence of a known malignancy, renal metastasis is much more common than renal cell cancer (RCC). The possible diagnosis of a renal mass in a patient with progressive primary disease is more likely metastases than RCC (7). However if the patient’s cancer is in remission, than the likelihood of RCC is increased. In our case, when recent PET scan was compared with the initial study both the number and the metabolic activity of the lesions were increased and this let us to consider metastases rather than a second primary. As in evaluating other renal masses, CT is the choice of modality that is most frequently used in the evaluation of a suspected metastatic renal lesion (2,4,5,8). When proper technique is used, the sensitivity and specificity may reach up to 100% and 95% respectively (9). A renal CT protocol consists of precontrast, arterial, corticomedullary, nephrographic and excretory phases (3,10,11). Initial noncontrast scans are used to detect calcifications and allow quantification of enhancement (3). The nephrographic phase is ideal to detect and characterize renal masses (11). The maximal and homogeneous parenchymal enhancement in this phase facilitates detection of renal masses, which typically does not enhance to the same degree as the renal parenchyma. In our case, a fully dedicated renal CT protocol was used. Although the cortical lesions in both kidneys were clearly seen, the number of lesions was underestimated and only the most prominent one in the lower pole of the left kidney was reported.

The most common CT appearance of renal metastases is bilateral, multifocal, small (< 3 cm), hypodense masses that do not commonly demonstrate hyperenhancement after contrast injection as this was the case in our patient (2,4). On the other hand, solitary lesion, necrosis, hyperenhancement after i.v. contrast injection, renal vein thrombosis and well-defined tumor margins support the diagnosis of RCC (2). 18F-FDG PET is specific in the diagnosis of renal masses, but its sensitivity depends on the size and location of the lesion (12). Due to the excreted radiopharmaceutical in the pelvicalyceal system and the lesions that do not accumulate 18F-FDG, the sensitivity and specificity of 18F-FDG PET/CT in detecting renal tumors are 60% and 100% respectively (13). However, with limited number of patients, Goldberg et al. suggested that 18F-FDG PET/CT might be successful in evaluating renal masses (14). In our case, the lesions were not reported in initial PET/CT and when we reviewed the images, although the size of the lesionswere not as large as those on the recent PET/CT, hypermetabolic lesions were also detected. The number and the size of the lesions were increased and they became more prominent in current study.

The missed renal cortical lesions on the PET/CT and contrast enhanced CT did not actually change the treatment approach in our case due to the advanced stage of the disease. However, in particular situations such as isolated kidney metastases these overlooked lesions might change the management of the disease.

In conclusion, the 18F-FDG uptake in the renal cortical region in a cancer patient should be evaluated carefully. Accumulation of 18F-FDG in minor calyces is interpreted as a normal finding in PET-CT. However, irregular 18F-FDG accumulation in renal cortex may be a sign of multiple kidney metastasis and every attempt should be performed to identify these lesions.
